# Complete mitochondrial genome sequence of nanday conure, *Aratinga nenday* (Psittaciformes: Psittacidae)

**DOI:** 10.1080/23802359.2019.1673253

**Published:** 2019-10-04

**Authors:** Da-Wei Liu, Yang Pan, Yong-Wu Zhou, Sen-Lin Hou

**Affiliations:** a Nanjing Forest Police College, Nanjing, PR China;; b Forensic Identification Center for Forest Police of the State Forest Bureau, Nanjing, PR China;; c Key Laboratory of State Forest and Grassland Administration Wildlife Evidence Technology, Nanjing, PR China;; d Ministry of Ecology and Environment, Nanjing Institute of Environmental Sciences, Nanjing, PR China

**Keywords:** Nanday conure, mitochondrial genome, *Aratinga nenday*

## Abstract

Here, we report the complete mitochondrial genome of *Aratinga nenday*. It was found to be a closed circular molecule of 16,983 bp, containing 22 transfer RNA genes, 13 protein-coding genes, 2 ribosomal RNA genes, and 1 non-coding control region. The overall base composition was 54.03% A + T and 45.973% G + C contents. The complete mtDNA of *A. nenday* and other 14 species were subjected to phylogenetic analysis by the neighbour-joining method and the Kimura 2-parameter model, which showed *A. nenday* to be a sister to the congeneric *Aratinga solstitialis*.

Nanday conure (*Aratinga nenday*) belongs to the family Psittacidae (Aves, Psittaciformes). This species is native to Argentina, Bolivia, Brazil, and Paraguay (Forshaw 2010), and inhabits forest and savanna (IUCN: Red List of Threatened Species 2019). Although this species is believed to have increased in population because of the creation of new suitable habitat areas owing to the ongoing habitat degradation (BirdLife International 2019), *A. nenday* was listed on CITES Appendix II in view of the associated wild animal trade.

In this study, we characterized and described the mitogenome of *A. nenday* to obtain basic genetic information of this species. A feather sample was collected from Shangqiu Zoo (34.46N°, 115.67E°) in Shangqiu, Henan Province, China. The voucher specimen (A-2019002) was stored in the Key Laboratory of Wildlife Evidence Technology State Forest and Grassland Administration. Genomic DNA was isolated using a kit (Takara, Beijing, China), and a set of primers was designed for PCR amplification. The amplified products were subjected to Sanger sequencing in order to obtain genome information. The complete mitochondrial genome of *A. nenday* was found to be a typical circular molecule composed of 16,983 bp (GenBank accession No. MK965540). It comprised 22 transfer RNA genes, 13 protein-coding genes, 2 ribosomal RNA genes (*rrnL*, *rrnS*), and 1 non-coding control region (D-loop). The gene order and organization of *A. nenday* was identical to that of other parrots (Eberhard and Wright 2016; Liu et al. 2019). The overall base composition of the mitogenome was relatively similar, with A + T content being 54.03% (A, G, C, and T was 30.30%, 14.03%, 31.94%, 23.74%, respectively).

The complete mitochondrial genome of *A. nenday* was then subjected to phylogenetic analysis along with that of 14 other parrot species recorded in the GenBank. A phylogenetic tree was constructed using the neighbour-joining method with 1000 bootstrap replicates and the Kimura 2-parameter model in MEGA 7.0 (Kumar et al. 2016). As a result, *A. nenday* was placed as sister to *Aratinga solstitialis* of the same genus (Figure 1). The *Aratinga* parrots were also closely related to *Ara severus* (Figure 1), which is distributed in Panama, Colombia, Brazil, and Peru (Forshaw 2010). This is the first report of the complete mitochondrial DNA of *A. nenday*, and the molecular data obtained could contribute to the future examination of evolutionary diversification of conures.

**Figure 1.**
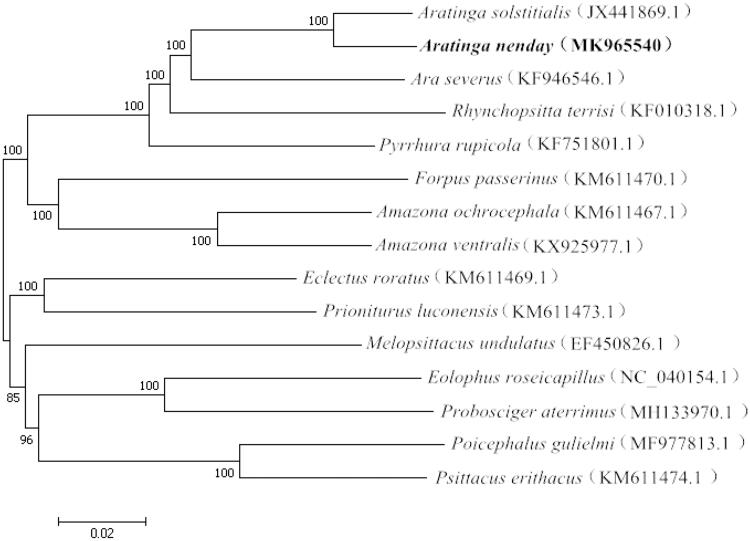
Neighbour-joining phylogenetic tree based on the complete mitogenomes of *A. nenday* and other 14 parrot species using MEGA 7.0.
